# The relationship between dietary supplement use in late pregnancy and birth outcomes: a cohort study in British women

**DOI:** 10.1111/j.1471-0528.2010.02549.x

**Published:** 2010-03-29

**Authors:** NA Alwan, DC Greenwood, NAB Simpson, HJ McArdle, JE Cade

**Affiliations:** a Nutritional Epidemiology Group, Centre for Epidemiology and Biostatistics, University of LeedsLeeds, UK; b Division of Biostatistics, Centre for Epidemiology and Biostatistics, University of LeedsLeeds, UK; c Department of Obstetrics and Gynaecology, University of LeedsLeeds, UK; d Rowett Institute of Nutrition and Health, University of AberdeenAberdeen, UK

**Keywords:** Birthweight, dietary supplements, pregnancy, pregnancy outcome, preterm birth

## Abstract

**Objective:**

To examine the relationship between dietary supplement use during pregnancy and birth outcomes.

**Design:**

A prospective birth cohort.

**Setting:**

Leeds, UK.

**Sample:**

One thousand two hundred and seventy-four pregnant women aged 18–45 years.

**Methods:**

Dietary supplement intake was ascertained using three questionnaires for the first, second and third trimesters. Dietary intake was reported in a 24-hour dietary recall administered by a research midwife at 8–12 weeks of gestation. Information on delivery details and antenatal pregnancy complications was obtained from the hospital maternity records.

**Main outcome measures:**

Birthweight, birth centile and preterm birth.

**Results:**

Reported dietary supplement use declined from 82% of women in the first trimester of pregnancy to 22% in the second trimester and 33% in the third trimester. Folic acid was the most commonly reported supplement taken. Taking any type of daily supplement during any trimester was not significantly associated with size at birth taking into account known relevant confounders. Women taking multivitamin-mineral supplements in the third trimester were more likely to experience preterm birth (adjusted OR = 3.4, 95% CI 1.2, 9.6, *P*= 0.02).

**Conclusions:**

Regular multivitamin–mineral supplement use during pregnancy, in a developed country setting, is not associated with size at birth. However, it appears to be associated with preterm birth if taken daily in the third trimester. The mechanism for this is unclear and our study’s findings need confirming by other cohorts and/or trials in developed countries.

## Introduction

Multivitamin–mineral supplements during pregnancy are becoming an attractive option considered by international agencies to improve the nutritional status of pregnant women in developing countries. They are considered relatively cheap, feasible and have the potential to improve maternal nutrition when administered through national antenatal programmes. However, dietary supplements are not subject to the same rigorous safety and efficacy standards as prescription medications.^1^ Their proposed use during pregnancy is supported by findings from several randomised controlled trials in developing country settings, where deficiency in micronutrients is more prevalent. Studies in Nepal, India, Indonesia, Guinea-Bissau and Tanzania have shown positive effects on adverse birth outcomes such as infant mortality and low birthweight.^2^–^6^ However, other trials in Nepal, Mexico and Zimbabwe have failed to demonstrate a significant effect on the incidence of low birthweight,^7^–^10^ and some have even demonstrated an increased risk of adverse outcomes.^10^,^11^ According to a Cochrane systematic review, there is currently insufficient evidence to suggest replacement of iron and folate supplementation with multiple micronutrient supplements and further research is needed to quantify the degree of maternal or fetal benefit and to assess the risk of excess supplementation and the potential for adverse interactions between the micronutrients.^12^

Although multivitamin supplements have been recommended for women who might become pregnant in some developed countries, such as the USA,^13^ there are few studies examining their effect on birth outcomes in developed countries, where there is likely to be a significant difference in women’s baseline nutrient status compared with developing countries. A randomised controlled trial in France showed significant positive effects for micronutrient supplementation versus placebo on the incidence of low birthweight.^14^ However, this study had a relatively small sample size of 100 women and a very small number of babies born with low birthweight. The supplements given in this study were iron-free and so differ from currently available over-the-counter multivitamin–mineral preparations for pregnant women. There was no difference detected in the oxidative stress parameters measured in the study between supplemented and unsupplemented women.

The Camden study on the impact of multivitamin supplementation on pregnancy was conducted in a disadvantaged urban setting in the USA.^15^ Risks of both low birthweight and preterm delivery were significantly reduced with supplement use in the first and second trimester. Analysis was restricted to data obtained by 28 weeks of gestation and the relationship between infant outcomes and supplement use in the third trimester of pregnancy was not reported.

We therefore analysed observational data collected for a large prospective cohort study, the Caffeine and Reproductive Health (CARE) birth cohort in Leeds, UK, to examine the relationship between supplement use during the first, second and third trimesters of pregnancy and two outcomes: birthweight and preterm delivery.

## Methods

### Participants

Women aged 18–45 years with low-risk pregnancies were prospectively recruited at 8–12 weeks of gestation from the Leeds Teaching Hospitals maternity unit between 2003 and 2006 as part of a multicentre prospective study into maternal diet and birth outcomes. The inclusion criteria and the methodology are described in detail elsewhere.^16^,^17^ All women participating in the study gave informed written consent and the study was approved by the Leeds West Local Research Ethics Committee.

### Assessment of diet and supplement use

Supplement use was ascertained throughout pregnancy using questionnaires in the first, second and third trimesters. The questionnaires were interviewer-administered during the first trimester (up to 12 weeks of gestation) and third trimester (from 28 weeks of gestation) and self-administered during the second trimester (13–27 weeks of gestation). In the third trimester, the interviews were performed retrospectively on a sub-sample of the cohort following a nested case–control design (*n* = 425) with a ratio of 2:1. The respondents were asked to report the type/brand, frequency and amount of all the dietary supplements they were using during each trimester. The questions were free text rather than multiple choice questions, asking participants to tick the type of supplements they were using to ensure all sources were covered. The supplement types were then coded during data entry. Dietary intake was reported in a 24-hour dietary recall administered by a research midwife at 8–12 weeks of gestation.

### Assessment of pregnancy outcomes

Information was obtained from the hospital maternity records on antenatal pregnancy complications and delivery details (gestational age at delivery, birthweight and sex of the baby). We analysed birthweight as the primary outcome measure in two forms: as a continuous variable in grams and as expressed on customised centile charts that took into account maternal height, weight, ethnicity and parity, and neonatal birthweight and sex.^18^ We examined preterm birth, defined as delivery at <37 weeks of gestation, as a secondary outcome measure.

### Statistical power calculations

Comparing birthweights between supplement users and nonusers within the first trimester, using the ratios of users to nonusers and standard deviations identified in the study, we had 80% power to detect a difference of 120 g; 90% power to detect a difference of 140 g, for *P*< 0.05. Within trimester 2, we had 80% power to detect a difference of <115 g; 90% power to detect a difference of 130 g. Within trimester 3, we had 85% power to detect a doubling of the prevalence of babies born less than the tenth centile (from 13% to 26%), and to detect a tripling of the preterm birth rate (from 5% to 15%) for a two-sided test at *P*< 0.05.

### Statistical methods

We performed univariable analyses using two-sample Student’s *t*-tests for continuous variables and chi-square tests for categorical variables. We used multiple linear regression for continuous outcomes and logistic regression for binary outcomes.

We performed unconditional logistic regression for small-for-gestational-age and preterm births, and general linear modelling for birthweight and customised birth centile using STATA version 10.^19^ Maternal age, height, weight, ethnicity and parity at booking and neonatal gestation at delivery and baby’s sex were taken into account in the definition for customised birth centile, and were adjusted for in the model for birthweight. We also made statistical adjustment for salivary cotinine levels, self-reported alcohol consumption, maternal age, maternal vegetarian diet, Index of Multiple Deprivation (IMD) score, the mother having a university degree, past history of miscarriage and long-term chronic illness in all models. The IMD 2007 combines a number of indicators (chosen to cover a range of economic, social and housing issues) into a single deprivation score for each small area in England. This allows each area to be ranked relative to one another according to their level of deprivation.^20^ Sensitivity analyses were performed taking into account clinical diagnosis of intrauterine growth restriction (IUGR) in the models.

With regards to the exposure of interest, analysis was performed using two groups; women who reported taking any type of daily supplements and those who specifically reported taking multivitamin–mineral supplements during pregnancy.

## Results

### Characteristics of women in supplement-taking and nonsupplement-taking groups

The total number of participants was 1274. All had information on supplement intake in the first and second trimester; 425 women had information on supplement intake in the third trimester. The proportion of pregnant women taking any form of daily supplements was 82%, 22% and 33% for the first, second and third trimesters, respectively (Table 1). Women who reported taking supplements at any stage of pregnancy were more likely to have a university degree and be vegetarian, and less likely to be smokers. They were less likely to be living in an area with an IMD score in the most deprived quartile. Women who reported taking daily supplements in the first and second trimester were more likely to be primiparous. However, there was no difference between primiparous and multiparous women in their use of supplements in the third trimester. There were also no differences between women who reported taking daily supplements at any stage in pregnancy from those who did not with regards to prepregnancy weight, ethnic origin or history of long-term illness. Out of the women who took daily supplements in the third trimester (*n* = 139), 94% (*n* = 131) also reported taking daily supplements in the first trimester of their pregnancy and 66% (*n* = 91) took daily supplements in their second trimester. Only five women who reported taking daily supplements in the third trimester had not taken supplements in the first or second trimester.

**Table 1:** Characteristics of women by whether they have reported taking any daily supplements in the first, second and third trimester, Leeds, UK, 2003–06

Characteristic Taking any daily supplements (*n*)	First trimester			Second trimester			Third trimester		
	Yes (1043)	No (231)	P*	Yes (274)	No (1000)	*P*	Yes (139)	No (286)	*P*
Age of mother (years) (mean, 95% CI**)	30 (30, 31)	29 (28, 29)	<0.001	31 (31, 32)	30 (29, 30)	<0.001	31 (30, 32)	29 (28, 30)	<0.001
Prepregnancy weight (kg) (mean, 95% CI)	67 (66, 68)	66 (65, 68)	0.4	66 (65, 68)	68 (67, 68)	0.2	67 (64, 69)	68 (66, 70)	0.4
Total energy intake (kcal) (mean, 95% CI)	2148 (2103, 2193)	2019 (1921, 2117)	0.02	2167 (2075, 2258)	2113 (2068, 2158)	0.3	2095 (1983, 2205)	2214 (2118, 2311)	0.1
Smoker at 12 weeks (%, 95% CI)	16 (14, 18)	28 (21, 37)	<0.001	11 (7, 15)	19 (17, 22)	0.002	15 (10, 23)	24 (19, 29)	0.04
IMD*** worst quartile (%, 95% CI)	28 (25, 31)	41 (35, 48)	<0.001	21 (16, 26)	33 (30, 36)	<0.001	18 (12, 25)	34 (29, 40)	0.002
European origin (%, 95% CI)	94 (92, 95)	92 (88, 95)	0.9	95 (91, 97)	93 (91, 95)	0.5	96 (91, 98)	96 (93, 98)	0.8
University degree (%, 95% CI)	43 (40, 46)	20 (15, 25)	<0.001	54 (48, 60)	35 (32, 38)	<0.001	51 (43, 60)	31 (26, 37)	<0.001
Vegetarian (%, 95% CI)	9 (7, 11)	7 (4, 12)	0.08	16 (12, 21)	7 (5, 9)	<0.001	15 (10, 22)	5 (2, 9)	0.003
Primigravida (%, 95% CI)	47 (44, 50)	40 (34, 47)	0.04	55 (49, 61)	44 (41, 47)	0.002	53 (45, 62)	50 (44, 56)	0.5
History of long term illness (%, 95% CI)	13 (11, 15)	10 (6, 15)	0.1	13 (9, 18)	13 (11, 15)	0.9	15 (10, 22)	16 (12, 21)	0.7
Average alcohol consumption more than 0.5 units/day throughout pregnancy (%, 95% CI)	28 (25, 31)	20 (14, 27)	0.03	28 (23, 34)	27 (24, 30)	0.7	27 (19, 35)	25 (20,31)	0.09
Past history of miscarriage (%, 95% CI)	23 (21, 33)	27 (21, 33)	0.3	27 (22, 32)	23 (20, 26)	0.2	21 (14, 29)	23 (18, 28)	0.8

* *P*-value using two-sample *t* test for continuous variables, chi-square test for categorical variables.

** Confidence interval.

*** Index of multiple deprivation.

### Dietary recall

Based on midwife-administered 24-hour recall dietary assessment at 8–12 weeks of gestation, women in our cohort had average dietary intakes from food above the reference nutrient intake values for most vitamins and minerals^21^ except vitamin D, iron, folate, selenium and iodine (Table 2). The table shows the mean intake in our cohort, the nutrient requirements for adult women plus the additional requirement recommended for consumption during pregnancy, and the proportion of the women in our cohort with dietary intakes above the recommended reference nutrient intake in pregnancy. The mean total energy intake was 2125 kcal/day (95% CI 2084, 2166).

**Table 2:** Average daily intakes of vitamins and minerals (from diet alone) based on 24-hour dietary recall at 8–12 weeks of pregnancy, Leeds, UK, 2003–06 (*n* = 1257)

Micronutrient	Mean (SD)	RNI*	Recommended increment to RNI during pregnancy**	Proportion of women with intakes above the pregnancy RNI (95% CI)
Thiamin (mg/day)	2.4 (7.7)	0.8	+0.1	85 (83, 87)
Riboflavin (mg/day)	1.7 (0.8)	1.1	+0.3	58 (55, 61)
Niacin (mg/day)	20 (10)	13	–	75 (72, 77)
Vitamin B6 (mg/day)	2.1 (1.0)	1.2	–	85 (82, 86)
Vitamin B12 (μg/day)	3.9 (3.7)	1.5	–	79 (77, 82)
Folate (μg/day)	257 (119)	200	+100	32 (29, 35)
Vitamin C (μg/day)	143 (129)	40	+10	75 (73, 78)
Vitamin A (μg retinol equivalent/day)	803 (665)	600	+100	45 (42, 48)
Vitamin D (μg/day)	2.5 (2.7)	–	10	2 (1, 3)
Vitamin E (mg/day)	7.9 (5.4)	–	****	–
Calcium (mg/day)	938 (471)	700	***	65 (62, 68)
Phosphorus (mg/day)	1344 (501)	550	***	98 (97, 99)
Magnesium (mg/day)	283 (112)	270	***	49 (46, 52)
Iron (mg/day)	11.5 (5.3)	14.8	***	20 (18, 23)
Zinc (mg/day)	8.6 (4.3)	7	***	59 (56, 62)
Copper (mg/day)	1.1 (0.6)	1.2	***	32 (29, 35)
Selenium (μg/day)	58 (37)	60	***	40 (38, 43)
Iodine (μg/day)	118 (82)	140	***	28 (24, 29)

* Reference nutrient intakes (RNI) for women aged 19–50 years in the UK.^21^

** Reference nutrient intakes for pregnant women.^21^

*** No increment.^21^

**** Safe intake – above 3 mg/day for women.^21^

### Type of supplements

Women reported taking 22 different types of supplements including folic acid, iron, combined folic acid–iron preparations, multivitamin–mineral preparations (six brands), evening primrose, cod liver oil, omega 3, vitamin C, vitamin B, vitamin D, vitamin E, vitamin A, calcium, zinc, magnesium and selenium preparations (Table 3). Folic acid was the most frequently reported daily supplement in the first trimester. Multivitamin–mineral supplements were the most frequently reported daily supplements in the third trimester.

**Table 3:** Number of women taking different types of supplements during pregnancy

Supplement	First trimester	Second trimester	Third trimester
Folic acid	845	51	2
Iron	8	21	29
Folic acid/iron	2	1	1
Multivitamin-mineral	293	177	79
Evening primrose	6	2	2
Cod liver oil	10	2	3
Omega 3 fish oil	11	12	9
Vitamin C	18	8	15
Vitamin B	7	0	2
Vitamin E	1	3	1
Vitamin A	0	0	1
Calcium	14	8	3
Zinc	7	1	1
Magnesium	2	0	0
Selenium	2	0	0

### Birth outcomes

Birthweight was known for 1259 babies. The mean birthweight was 3439 g (95% CI 3397, 3461). 4.4% weighed <2500 g (*n* = 55). 13% (*n* = 166) weighed less than the tenth centile, 8% (*n* = 99) less than the fifth centile and 5% (*n* = 65) less than the third centile. 9% (*n* = 118) weighed more than the 90th centile. Out of the 1234 pregnancies with information on gestational age, 55 (4.5%) delivered before 37 weeks of gestation.

### Relationship between supplement taking and birthweight

Using a multiple linear regression model, taking any type of daily supplement during the first, second or third trimester of pregnancy was not associated with the customised birth centile as a measure of birth size (adjusted difference 2.7, 95% CI: 2.5, 7.8, *P*= 0.3 for the first trimester; 3.2, 95% CI: 0.9, 7.4, *P*= 0.1 for the second trimester; and 0.5, 95% CI: 6.0, 7.0, *P*= 0.9 for the third trimester) when adjusting for cotinine levels, self-reported alcohol intake, IMD group, having a university degree (39%), mother being a vegetarian (9%), history of long-term chronic illness (13%) and past history of miscarriage (24%).

Using birthweight in grams as an outcome, and adjusting for the above factors as well as maternal age, height, ethnicity, prepregnancy weight, parity, gestational age and baby’s sex, there was also no relationship between taking daily supplements at any stage in pregnancy and birthweight (adjusted difference 6 g, 95% CI: 70, 82, *P*= 0.9 for the first trimester, 24 g, 95% CI: 36, 83, *P*= 0.4 for the second trimester, and −7 g, 95% CI: 106, 91, *P*= 0.9 for the third trimester).

When we looked at taking particular types of supplements, taking a daily multivitamin–mineral preparation at any stage in pregnancy was not associated with size at birth using the continuous outcomes of birthweight in grams and customised birth centile, as well as the binary outcome of small-for-gestational-age (less than tenth centile) (Table 4). It was not associated with having a baby weighing less than the third centile (adjusted OR = 1.5, 95% CI 0.8, 2.7, *P*= 0.3 for the first trimester, 1.2, 95% CI 0.5, 2.6, *P*= 0.7 for the second trimester, 1.6, 95% CI 0.7, 3.7, *P*= 0.3 for the third trimester). There were no associations with having a baby weighing less than the fifth centile or more than the 90th centile. In addition, taking iron-containing supplements at any stage in pregnancy was not associated with size at birth (data not shown).

**Table 4:** The relationship between maternal multivitamin-mineral supplement use during pregnancy and birth outcomes, Leeds, UK, 2003–06

Daily multivitamin–mineral supplements	Unadjusted difference (95% CI)	*P* value	Adjusted difference (95% CI)	*P* value
**Birthweight (g)**				
First trimester	30.0 (−45.7, 105.7)	0.5	*16.9 (−42.3, 75.8)	0.7
Second trimester	38.4 (−53.6, 130.5)	0.4	29.4 (−43.0, 101.5)	0.3
Third trimester	−29.1 (−179.9, 121.6)	0.7	−50.4 (−168.7, 67.9)	0.4
**Customised birth centile**				
First trimester	3.6 (−0.2, 7.5)	0.06	**1.8 (−2.3, 5.9)	0.4
Second trimester	5.1 (0.4, 9.7)	0.04	3.3 (−1.8, 8.3)	0.3
Third trimester	1.2 (−6.5, 8.8)	0.8	−2.3 (−10.3, 5.7)	0.8
**Small for gestational age (<10th centile)**				
First trimester	1.0 (0.6, 1.4)	0.8	1.3 (0.8, 1.9)	0.3
Second trimester	0.9 (0.5, 1.4)	0.6	1.1 (0.7, 1.9)	0.7
Third trimester	0.7 (0.4, 1.3)	0.3	0.9 (0.5, 1.7)	0.8
**Preterm birth (<37 weeks)**				
First trimester	0.9 (0.5, 1.8)	0.8	***1.3 (0.6, 2.7)	0.5
Second trimester	1.0 (0.5, 2.2)	0.9	1.8 (0.8, 4.1)	0.2
Third trimester	1.8 (0.8, 4.4)	0.2	3.4 (1.2, 9.6)	0.02

* Adjusted for gestational age, baby’s sex, maternal age, height, prepregnancy weight, ethnicity, parity, salivary cotinine levels, self-reported alcohol intake, past history of miscarriage, long-term chronic illness, index of multiple deprivation (IMD) score, educational attainment and maternal vegetarian diet in a multiple linear regression model.

** Adjusted for salivary cotinine levels, self-reported alcohol intake, past history of miscarriage, long-term chronic illness, IMD score, educational attainment and maternal vegetarian diet in a multiple linear regression model.

*** Adjusted for salivary cotinine levels, self-reported alcohol intake, maternal age, maternal vegetarian diet, ethnicity, baby’s sex, parity, IMD score, educational attainment, past history of miscarriage and long-term chronic illness in an unconditional logistic regression model.

### Relationship between supplement taking and preterm birth

We used a logistic regression model to examine the relationship between the risk of preterm birth and patterns of supplement-taking during pregnancy adjusting for salivary cotinine levels, self-reported alcohol intake, vegetarian diet, ethnicity, maternal age, baby’s sex, parity, IMD score, having a university degree, past history of miscarriage and long-term chronic illness. Any type of daily supplement-taking during the third trimester was associated with an increase in the risk of preterm birth (adjusted OR 3.0, 95% CI 1.2, 7.4, *P*= 0.02). This relationship was not statistically significant for supplement-taking in the second trimester (adjusted OR 1.6, 95% CI 0.8, 3.2, *P*= 0.2) and was marginally significant in the first trimester, although confidence intervals were wide (adjusted OR 4.3, 95% CI 1.0, 18.2, *P*= 0.05).

Taking multivitamin–mineral supplement preparations during the third trimester was also associated with an increased risk of preterm birth (adjusted OR 3.4, 95% CI 1.2, 9.6, *P*= 0.02). This relationship was not statistically significant in the first or second trimester (Table 4). When looking at any iron-containing supplement, the relationship remained significant only for supplement-taking in the third trimester (adjusted OR 3.0, 95% CI 1.2, 7.6, *P*= 0.02).

### Sensitivity analyses

In addition, we adjusted for the clinical diagnosis of IUGR detected by ultrasound scan during pregnancy and documented in the maternity notes, in the relationship between taking a multivitamin–mineral supplement preparation and both birthweight and preterm birth. The risk of preterm birth when taking supplements in the third trimester (adjusted OR 3.5, 95% CI 1.2, 10.0, *P*= 0.02) remained broadly unchanged.

To take into account the possibility that the pattern of multivitamin–mineral supplement use is influenced by previous adverse birth outcomes, we also performed the same analysis separately by parity. In primiparous women, the adjusted OR for the relationship between taking multivitamin–mineral supplement in the third trimester and preterm birth was 5.4 (95% CI 1.3, 22.7, *P*= 0.02). In multiparous women, the adjusted OR was 3.7 (95% CI 0.5, 29.4, *P*= 0.2). However, numbers were small with resulting wide confidence intervals.

## Discussion

Our results show that taking daily multivitamin–mineral supplements during any stage in pregnancy is not associated with lower birthweight. However, taking multivitamin–mineral supplements in the third trimester is associated with a three-fold increased risk of preterm birth after adjustment for smoking, alcohol intake and other relevant maternal and socioeconomic factors. This effect seems more pronounced in primiparous women. Although the number of women taking supplements in the third trimester was considerably less than that for the first two trimesters, there was enough power with the nested case–control design to detect an odds ratio of 3 for the preterm birth outcome. However, this study is observational so causality cannot be inferred from the findings. As we did not have information on iatrogenic preterm birth, it is possible that some women knew that they were at risk of preterm birth and that this knowledge initiated physician or patient-led supplementation. However, in our study, only five women who reported taking daily supplements in the third trimester, did not take supplements in the first and second trimester. None of these five women had a preterm birth.

Because this is not a randomised controlled trial, we cannot rule out the possibility that residual confounding may be contributing to this apparent association. There may be unmeasured confounders resulting in the apparent negative relationship between multivitamin supplement taking in the third trimester and preterm birth. However, we have adjusted for most factors known to confound this relationship. The possibility that supplement use may be influenced by a woman knowing that the baby is not growing as would be expected is taken into account by adjusting for the clinical diagnosis of IUGR, as extracted from the pregnancy medical notes, in a sensitivity analysis.

We have considered the potential that previous poor pregnancy outcome may influence the mother’s decision to take supplements in subsequent pregnancies and therefore, adjusted for past history of miscarriage in the main models and performed the analysis separately for primiparous and multiparous women in a sensitivity analysis. The hypothesis is that women with previous adverse pregnancy outcomes would be more likely to take supplements as well as to experience adverse outcomes in their subsequent pregnancies. This would confound the relationship between supplement-taking in the third trimester and preterm birth. However, we found this relationship to be more pronounced in primiparous women. This means that the effect is not influenced by previous birth outcomes.

The use of multivitamin–mineral supplements in our cohort was restricted mainly to two pregnancy-specific brands. Both brands included folate and vitamin C exceeding the current recommended minimum during pregnancy (Table 2). One of the brands had the additional components of B-carotene, vitamin K, selenium and iodine as well as higher doses of vitamins E, B1, B6 and B12 and zinc (at least double) compared with the other main brand. Women in our cohort were receiving adequate amounts of these micronutrients from their diet alone, as assessed by the 24-hour dietary recall (Table 2), confirming the inverse supplement hypothesis, that women who least need supplements are most likely to take them.^22^

Other studies have suggested potential adverse effects of some supplements, specifically those containing antioxidant vitamins such as vitamins C and E, on pregnancy outcome when taken by women with adequate dietary micronutrient intake. Smedts *et al.*,^23^ in a case–control study of offspring with congenital heart disease, found that periconceptual use of vitamin E supplements with high dietary intake of the same vitamin was associated with up to nine-fold increase in the risk of congenital heart disease. Another study found that use of supplements for vitamins C and E was associated with an increased risk of premature rupture of membranes.^24^ Unfortunately, this information was not recorded in our study. In a randomised controlled trial to assess the effect of vitamin E and C supplementation during pregnancy on the incidence of pre-eclampsia, Poston *et al.*^25^ found that more low birthweight babies were born to women who took these antioxidants than to controls. A recent-meta-analysis of seven studies concluded that combined vitamin C and E supplementation had no potential benefit in the improvement of maternal and neonatal outcome and increased the risk of gestational hypertension in women at risk of pre-eclampsia.^26^

It is well established that there are significant interactions between micronutrients and their metabolism. It has been shown in rats, for example, that copper deficiency during pregnancy can result in reduced iron status and vice versa, and that copper overload induces iron overload, by interfering with the iron regulatory mechanism.^27^,^28^ Others have demonstrated interactions between iron and zinc.^29^ During the third trimester, fetal growth is at its most rapid. The fetus not only needs minerals to sustain its growth, it is also a stage when the fetal liver builds up stores for the immediate postnatal period. A reduction in mineral availability, by interactions between the nutrients in the maternal gut or liver or in the placenta itself, may result in adverse outcomes for the baby.

The pattern of dietary supplement use in our cohort, with most women taking supplements (mainly folic acid) in the first trimester, is expected because there is no national recommendation in England for routine supplement-taking during pregnancy apart from folic acid in the first trimester and vitamin D for pregnant women in ‘high-risk’ groups.^30^ There is no national recommendation to take multivitamin and mineral supplements at any stage during pregnancy. However, they are readily available over-the-counter and are heavily promoted to expectant mothers. Health value and susceptibility to illness are major predictors of supplement use by women, with dietary supplements acting as an insurance against possible ill health.^31^

### Implications for research

Most previous trials and observational studies in developed country settings have looked at the effect of taking multivitamin supplementation in early pregnancy on maternal and birth outcomes. More research is needed into the effect of taking multivitamin–mineral supplements in late pregnancy on birth outcomes in relatively well-nourished populations. Larger cohort studies are required to examine this association in detail and to validate the findings of this study. Results from our cohort also suggest that a trial in a developed country setting is needed to weigh the possible benefits and harms of policies recommending supplementation or restriction of supplementation.

### Implications for clinical practice

The study findings suggest that clinicians and midwives should be cautious when recommending over-the-counter multivitamin supplements to women in late pregnancy. As in any clinical situation, they should weigh the potential risks and benefits when considering prescribing such supplements during the third trimester of pregnancy. The type of supplement recommended/prescribed should be more focused on the specific vitamin/mineral deficiency the woman has. Although the negative relationship between multivitamin supplement-taking in the third trimester and preterm birth needs to be investigated further, this study did not show any positive effect on birthweight and gestational age when these supplements are taken at any stage in pregnancy.

## Conclusion

In this study, the use of multivitamin and mineral supplement preparations during the third trimester in pregnancy was associated with an increased risk of preterm delivery, and was not associated with birthweight, small- or large-for-gestational-age, at any stage in pregnancy. These findings suggest that, at least in micronutrient-replete mothers, caution must be exercised when recommending multivitamin–mineral supplements in late pregnancy. This is an observational prospective study offering weaker causal evidence than a randomised controlled trial. However, in the absence of a trial in a developed country setting, this study makes a useful contribution to the research evidence in this area. The findings generate a concern regarding multivitamin supplement use in late pregnancy that needs to be investigated by other studies.

## References

[1] Gardiner PM, Nelson L, Shellhaas CS, Dunlop AL, Long R, Andrist S (2008). The clinical content of preconception care: nutrition and dietary supplements. Am J Obstet Gynecol.

[2] Osrin D, Vaidya A, Shrestha Y, Baniya RB, Manandhar DS, Adhikari RK (2005). Effects of antenatal multiple micronutrient supplementation on birthweight and gestational duration in Nepal: double-blind, randomised controlled trial. Lancet.

[3] Gupta P, Ray M, Dua T, Radhakrishnan G, Kumar R, Sachdev HPS (2007). Multimicronutrient supplementation for undernourished pregnant women and the birth size of their offspring: a double-blind, randomized, placebo-controlled trial. Arch Pediatr Adolesc Med.

[4] The Supplementation with Multiple Micronutrients Intervention Trial (SUMMIT) Study Group (2008). Effect of maternal multiple micronutrient supplementation on fetal loss and infant death in Indonesia: a double-blind cluster-randomised trial. Lancet.

[5] Kaestel P, Michaelsen KF, Aaby P, Friis H (2005). Effects of prenatal multimicronutrient supplements on birth weight and perinatal mortality: a randomised, controlled trial in Guinea-Bissau. Eur J Clin Nutr.

[6] Fawzi WW, Msamanga GI, Urassa W, Hertzmark E, Petraro P, Willett WC (2007). Vitamins and perinatal outcomes among HIV-negative women in Tanzania. N Engl J Med.

[7] Christian P, Khatry SK, Katz J, Pradhan EK, LeClerq SC, Ram Shrestha S (2003). Effects of alternative maternal micronutrient supplements on low birth weight in rural Nepal: double blind randomised community trial supplements. BMJ.

[8] Friis H, Gomo E, Nyazema N, Ndhlovu P, Krarup H, Kaestel P (2004). Effect of multimicronutrient supplementation on gestational length and birth size: a randomized, placebo-controlled, double-blind effectiveness trial in Zimbabwe. Am J Clin Nutr.

[9] Ramakrishnan U, Gonzalez-Cossio T, Neufeld LM, Rivera J, Martorell R (2003). Multiple micronutrient supplementation during pregnancy does not lead to greater infant birth size than does iron-only supplementation: a randomized controlled trial in a semirural community in Mexico. Am J Clin Nutr.

[10] Christian P, Darmstadt G, Wu L, Khatry S, Leclerq S, Katz J (2008). The effect of maternal micronutrient supplementation on early neonatal morbidity in rural Nepal: a randomised, controlled, community trial. Arch Dis Child.

[11] Roberfroid D, Huybregts L, Lanou H, Henry M-C, Meda N, Menten J (2008). Effects of maternal multiple micronutrient supplementation on fetal growth: a double-blind randomized controlled trial in rural Burkina Faso. Am J Clin Nutr.

[12] Haider B, Bhutta Z (2006). Multiple-micronutrient supplementation for women during pregnancy. Cochrane Database Syst Rev.

[13] Willett WC, Stampfer MJ (2001). What vitamins should I be taking, doctor?. N Engl J Med.

[14] Hininger I, Favier M, Arnaud J, Faure H, Thoulon JM, Hariveau E (2004). Effects of a combined micronutrient supplementation on maternal biological status and newborn anthropometrics measurements: a randomized double-blind, placebo-controlled trial in apparently healthy pregnant women. Eur J Clin Nutr.

[15] Scholl TO, Hediger ML, Bendich A, Schall JI, Smith WK, Krueger PM (1997). Use of Multivitamin/mineral prenatal supplements: influence on the outcome of pregnancy. Am J Epidemiol.

[16] CARE Study Group (2008). Maternal caffeine intake during pregnancy and risk of fetal growth restriction: a large prospective observational study. BMJ.

[17] Boylan SM, Cade JE, Kirk SFL, Greenwood DC, White KLM, Shires S (2008). Assessing caffeine exposure in pregnant women. Br J Nutr.

[18] Gardosi J (2004). Customised fetal growth standards: rationale and clinical application. Semin Perinatol.

[19] Stata Corporation. Stata statistical software: Release 10 (2007).

[20] Department for Communities and Local Government. Indices of deprivation (2009). http://www.communities.gov.uk/communities/neighbourhoodrenewal/deprivation/deprivation.

[21] Department of Health (1991). Dietary Reference Values. A Guide.

[22] Conner M, Kirk S, Cade J, Barrett J (2003). Environmental influences: factors influencing the decision to use dietary supplements in women. J Nutr.

[23] Smedts HPM, Vries JHd, Rakhshandehroo M, Wildhagen MF, Verkleij-Hagoort AC, Steegers EA (2009). High maternal vitamin E intake by diet or supplements is associated with congenital heart defects in the offspring. BJOG.

[24] Spinnato IiJA, Freire S, Pinto e Silva JL, Rudge MVC, Martins-Costa S, Koch MA (2008). Antioxidant supplementation and premature rupture of the membranes: a planned secondary analysis. Am J Obstet Gynecol.

[25] Poston L, Briley AL, Seed PT, Kelly FJ, Shennan AH (2006). Vitamin C and vitamin E in pregnant women at risk for pre-eclampsia (VIP trial): randomised placebo-controlled trial. Lancet.

[26] Rahimi R, Nikfar S, Rezaie A, Abdollahi M (2009). A meta-analysis on the efficacy and safety of combined vitamin C and E supplementation in preeclamptic women. Hypertens Pregnancy.

[27] Gambling L, Anderson HS, McArdle HJ (2008). Iron and copper, and their interactions during development. Biochem Soc Trans.

[28] Fosset C, Danzeisen R, Gambling L, McGaw BA, McArdle HJ (2009). Cu loading alters expression of non-IRE regulated, but not IRE regulated, Fe dependent proteins in HepG2 cells. J Inorg Biochem.

[29] Kelleher SL, Lonnerdal B (2006). Zinc supplementation reduces iron absorption through age-dependent changes in small intestine iron transporter expression in suckling rat pups. J Nutr.

[30] National Institute for Clinical Excellence (NICE) (2008). Antenatal Care: Routine Care for the Healthy Pregnant Woman.

[31] Conner M, Kirk SFL, Cade JE, Barrett JH (2001). Why do women use dietary supplements? The use of the theory of planned behaviour to explore beliefs about their use. Soc Sci Med.

